# Old cortex, new contexts: re-purposing spatial perception for social cognition

**DOI:** 10.3389/fnhum.2013.00645

**Published:** 2013-10-08

**Authors:** Carolyn Parkinson, Thalia Wheatley

**Affiliations:** Department of Psychological and Brain Sciences, Dartmouth CollegeHanover, NH, USA

**Keywords:** exaptation, neural reuse, social neuroscience, metaphor, spatial cognition, perspective taking, social distance, posterior parietal cortex

## Abstract

Much of everyday mental life involves information that we cannot currently perceive directly, from contemplating the strengths of friendships to reasoning about the contents of other minds. Despite their primacy to everyday human functioning, and in particular, to human sociality, the mechanisms that support abstract thought are poorly understood. An explanatory framework that has gained traction recently in cognitive neuroscience is exaptation, or the re-purposing of evolutionarily old circuitry to carry out new functions. We argue for the utility of applying this concept to social cognition. Convergent behavioral and neuroscientific evidence suggests that humans co-opt mechanisms originally devoted to spatial perception for more abstract domains of cognition (e.g., temporal reasoning). Preliminary evidence suggests that some aspects of social cognition also involve the exaptation of substrates originally evolved for processing physical space. We discuss the potential for future work to test more directly if cortical substrates for spatial processing were exapted for social cognition, and in so doing, to improve our understanding of how humans evolved mechanisms for navigating an exceptionally complex social world.

## EXAPTATION AND HUMAN COGNITION

Our thoughts often include information outside of the current sensory environment, from imagined futures to the contents of other minds. However, the mechanisms supporting abstract cognition remain poorly understood. An explanatory framework that has gained traction recently (Gallese and Lakoff, 2005; Dehaene and Cohen, 2007; Anderson, 2010) involves exaptation: co-opting existing morphological features for novel functions (Gould and Vrba, 1982). New cognitive capacities may have emerged over the course of evolution when brain regions originally devoted to specific functions were repurposed and recombined in novel ways to process additional kinds of information (Anderson, 2010). Analogous cortical recycling processes may occur during development whereby cultural inventions co-opt circuitry evolved for older aspects of cognition (Dehaene and Cohen, 2007). Exaptation and cortical recycling provide plausible neural bases for proposals that representational resources originally devoted to space were co-opted to process more abstract information (e.g., Boroditsky, 2011).

If our ability to reason about abstract concepts resulted from evolutionary “tinkering” (Jacob, 1977) with neural mechanisms originally developed for operating on physical space, then demanding that these mechanisms handle conflicting inputs pertaining to their new and old functions simultaneously should create response conflict. Further, evidence from clinical and neuroimaging studies should suggest shared substrates for these functions. Both kinds of evidence are accumulating with respect to several domains of abstract cognition, most widely in studies relating temporal and numerical processing to spatial cognition (Hubbard et al., 2005; Bonato et al., 2012). Here, we highlight the potential for our understanding of the mechanisms underlying abstract social cognition to benefit from a similar approach, and review evidence that these mechanisms may be best understood in terms of the kinds of computations (e.g., distance judgments, perspective taking), rather than the domains of knowledge, that they involve.

## SOCIALITY AND HUMAN BRAIN EVOLUTION

Humans come into the world seemingly hardwired to detect and connect with other minds (Wheatley et al., 2012), and maintaining this predisposition is closely tied to healthy development (Pavlova, 2012). Effectively perceiving and interpreting social cues is particularly crucial for humans, who must navigate an exceptionally flexible system of relationships with conspecifics (Fiske, 1991). The ability to meet the intensive computational demands of humans’complex social environment (e.g., forging alliances, sharing intentions, tactical deception; Harcourt, 1988, 1989; Tomasello et al., 2005) is thought to have been a driving force for cortical expansion during evolution (Dunbar, 1998). Consistent with this hypothesis, feats of social cognition presumed to be uniquely human, such as sharing intentions (Tomasello et al., 2005) and representing others’beliefs (representational theory of mind, RTOM; Call and Tomasello, 2008) involve cortical areas that underwent the most evolutionary expansion (e.g., lateral posterior parietal cortex, PPC; Van Essen et al., 2001; particularly the temporoparietal junction, TPJ; Saxe, 2006; Redcay et al., 2010). These aspects of social cognition (e.g., RTOM) tend to involve information that cannot be perceived directly (e.g., false beliefs), and are functionally (Gobbini et al., 2007) and structurally (Parkinson and Wheatley, 2012) dissociable from older social processes (e.g., motor resonance), suggesting they either involve entirely new structures or structures previously devoted to non-social functions. Gould and Vrba (1982) suggested that exaptation of complex traits would likely be followed by secondary adaptations to further support new functions. Consistent with PPC circuitry evolved for dealing with space having been exapted, then expanded, to support abstract social cognition, the PPC has an evolutionarily old role in spatial perception (it encodes space in our distant relatives, e.g., rats; Nitz, 2006), processes both social and spatial information in humans and other primates (Yamazaki et al., 2009), and has expanded (Van Essen et al., 2001) and formed new connections (Mantini et al., 2013) in humans as it came to support evermore abstract aspects of social cognition. Recent computational modeling experiments support the notion that human brain expansion was driven by the cognitive demands of human sociality (Dávid-Barrett and Dunbar, 2013). Importantly, large brain size has a great metabolic cost; the human brain accounts for 2% of body mass but requires 20% of the energy that we consume (Clark and Sokoloff, 1999). In order to outweigh the considerable metabolic cost of the larger brain that they require, the cognitive mechanisms supporting human sociality must have conferred substantial adaptive benefits.

However, compared to other domains of abstract cognition (e.g., mathematics; Hubbard et al., 2005), little is known about how social forms of abstract cognition (e.g., representing beliefs or one’s place in a social network) relate to evolutionarily older aspects of cognition. This may be due to several factors. First, compared to cognitive neuroscience, social cognitive neuroscience is a young field (Ochsner, 2007); many aspects of social cognition have simply been studied less extensively than other aspects of cognition. Second, early social cognitive neuroscience research often assumed a modular view of the brain (Bergeron, 2007), and involved searching for encapsulated brain areas devoted to processing particular contents (Kihlstrom, 2010). If one understands an aspect of cognition to be supported by a domain-specific module, attempting to relate that aspect of cognition to other mental phenomena may not be considered a particularly worthwhile endeavor. More recently, brain areas (e.g., TPJ; fusiform face area) previously implicated in various facets of social information processing (e.g., RTOM; face perception) have been found to perform similar operations (e.g., reorienting attention, Mitchell, 2008; visual object encoding, Hanson et al., 2004) on diverse contents. Consistent with the suggestion that social cognition and physical perception involve common computations (Zaki, 2013), the functional significance of brain areas involved in social cognition may often be best characterized in terms of the operations they perform across multiple domains of information.

## LINGUISTIC MAPPINGS BETWEEN ABSTRACT COGNITION AND SPATIAL PERCEPTION

One window into the cognitive operations supporting abstract thought is the language we use to describe them (Lakoff and Johnson, 1980). The spatialization of form hypothesis (Lakoff, 1987) specifically highlights the widespread use of spatial words (e.g., “outside,” “far”) to describe conceptual relations, suggesting that spatial schemata structure mental representations. Abstract relations may be represented in terms of space because unlike spatial relationships, they must be imagined rather than observed (Evans, 2006). We can observe two people sitting close together, gaze direction, or moving a vehicle forward, but can only imagine the closeness of a friendship, a belief, or moving a meeting forward (Casasanto et al., 2010). In this view, phrases like “close friendship” or “far from the truth” are not mere figures of speech, but rather, figures of thought that reveal the structure of mental representations (Lakoff, 1986). The extent to which representational overlap between space and abstract domains results from exaptation during evolution, metaphoric structuring acquired during development, or some combination of these processes, remains an open question. With respect to social processing, the recruitment of brain areas involved in reorienting visual attention (TPJ) while congenitally blind individuals perform RTOM tasks (Bedny et al., 2009) suggests that functional overlap between social and visuospatial processes may be an innately predisposed result of evolutionary exaptation that is now reflected in linguistic metaphors for mentalizing (e.g., “Try to see things from my point of view”).

The domain of abstract cognition that has been studied most extensively in terms its relation to space is time. Cross-linguistic studies indicate that people around the world use spatial language to describe time (Boroditsky, 2011); the intuition to represent time analogously to space may be evolutionarily predisposed. Do all languages employ spatial language to describe social relationships (e.g., “close friend”) and RTOM? Are mappings consistent across languages? Some cross-linguistic variability exists in spatiotemporal metaphors, but certain mappings (future = forward) are nearly ubiquitous, likely due to shared aspects of human physiology and experience. Similarly, some English spatial metaphors for social relationships (familiarity = closeness) may stem from the tendency to give personal space to others based on the “closeness” of relationships (Hayduk, 1983). To our knowledge, metaphoric mappings between spatial and social relationships or between visuospatial and social perspective taking have not been subjected to exhaustive cross-linguistic analysis. Thus, whether or not humans around the world use space to structure mental representations of the magnitude and traversal of social distances remains an open question.

## BEHAVIORAL EVIDENCE FOR MAPPINGS BETWEEN ABSTRACT COGNITION AND SPATIAL PERCEPTION

Behavioral mappings between space and abstract cognition have been most extensively studied with respect to time and number. Number and space are associated implicitly; according to the spatial numerical association of response codes (SNARC) effect, people are faster to respond regarding small numbers on the left side of space, and large numbers on the right side of space, even for tasks unrelated to magnitude (Dehaene et al., 1993). Similar associations have been documented between number and elevation (Pecher and Boot, 2011; Lugli et al., 2013). Representational overlap between space and number appears to comprise a universal human intuition (Dehaene et al., 2008), and can be documented outside of the laboratory. When thinking about numbers, more than 10% of individuals report automatically accessing mental “number forms” consisting of spatial layouts (Seron et al., 1992). It has even been suggested that on the scale of motoric action, time, space, and quantity are processed by an analog magnitude system (Walsh, 2003), which was co-opted to process discrete number (Bueti and Walsh, 2009).

Stimulus-response compatibility codes also exist for time (Ishihara et al., 2008; Sell and Kaschak, 2011). Additionally, people tend to spontaneously sway forward while imagining the future and backward while imagining the past, suggesting that representations of movement through space are automatically activated during imagined movement through time (Miles et al., 2010). Monkeys (Merritt et al., 2010) and infants without exposure to relevant linguistic or sensorimotor mappings (Srinivasan and Carey, 2010) exhibit representational overlap between spatial extent and temporal duration (but not all magnitudes), suggesting that spatiotemporal mappings originate from common processing mechanisms, independently of sensorimotor grounding or linguistic correspondences.

Social and spatial information are also behaviorally associated. Visual perspective taking and mentalizing abilities are positively correlated (Flavell et al., 1986; Hamilton et al., 2009). People readily convert judgments of social compatibility into physical distances (Yamakawa et al., 2009). Words characterizing close social distances (e.g., “us,” “friend”) are associated with close locations, and words characterizing remote social distances (e.g., “them,” “enemy”) are associated with far spatial locations (Bar-Anan et al., 2007). Additionally, consistent with the suggestion that out-group members are construed as being physically distant from oneself (except following threat, Xiao and Van Bavel, 2012), Jones et al. (1981) found that out-group members are rated as more homogenous (i.e., having a narrower range of personal characteristics) than in-group members. Similarly, powerful individuals, who see themselves as exceptionally distinctive, construe others as exceptionally distant and homogenous (Fiske, 1993; Lee and Tiedens, 2001). It may be parsimonious to represent social and spatial distances analogously: Construal level theory of psychological distance (Liberman and Trope, 2008) posits that spatial, temporal and social egocentric distance share a common psychological meaning – distance from the self in the here and now.

Although extant research highlights a possible relationship between mental representations of social and spatial information, more research is needed to explore this possibility, and address several remaining questions, such as: is there a hierarchy of egocentric psychological distance domains, in which some are more primary than others? Do we spontaneously access representations of moving through space when traversing “social” distances or perspective taking, like during mental time travel? Are spatial representations activated explicitly when thinking about social relationships in everyday life, as they are for many individuals when thinking about numbers? Exploring questions like these will lead to an improved understanding of the mechanisms involved in abstract social cognition.

## NEUROSCIENTIFIC EVIDENCE FOR MAPPINGS BETWEEN ABSTRACT COGNITION AND SPATIAL PERCEPTION

If spatial processing were repurposed for abstract cognition, one would expect overlapping neural substrates. Past research suggests that PPC systems for sensorimotor control and cognition largely overlap (Creem-Regehr, 2009). As the PPC expanded in size over the course of human evolution (Van Essen et al., 2001), it appears to have expanded in function as well, leading to suggestions that mechanisms originally devoted to representing peripersonal space were repurposed to perform analogous operations on new contents. According to this theory, mechanisms previously dedicated to representing spatial information about the current sensory environment were first co-opted to represent simulations of peripersonal space in the past and future to support episodic memory and prospection, and later, to represent information in increasingly abstract frames of reference (Yamazaki et al., 2009). A growing body of neuroimaging and neuropsychological evidence suggests that representations of spatial and abstract information, including aspects of social cognition, are associated in the PPC.

Functional magnetic resonance imaging (fMRI) studies in humans implicate the PPC in representing perceptual, temporal, social and conceptual frames of reference (Yamazaki et al., 2009). Importantly, most of these results are based on overlapping activations from univariate contrasts, which could reflect shared neural codes or nearby but distinct codes for different kinds of information (Peelen and Downing, 2007). Multivariate pattern analysis (MVPA), which compares distributed patterns of activity between experimental conditions, rather than regionally smoothed and averaged responses, may better characterize brain regions’representational contents (**Figure 1**). The few studies that have used MVPA to compare spatial and abstract cognition in the PPC support the suggestion that representations of spatial information “scaffold” those of more abstract information. A pattern classifier trained only to distinguish PPC responses to leftward vs. rightward saccades can distinguish mental addition from subtraction (Knops et al., 2009). Additionally, position and valence words can be decoded by a classifier trained only on patterns of PPC activity corresponding to visual elevation (Quadflieg et al., 2011). Because MVPA can reveal information about underlying cognitive structures (**Figure 1**), this approach will be valuable in elucidating whether use of spatial language in describing abstract social concepts reflects true representational similarities or linguistic bottlenecks that push people to use metaphors in the absence of adequate domain-specific terminology (such bottlenecks have been demonstrated in olfaction; Yeshurun and Sobel, 2010).

**FIGURE 1:**
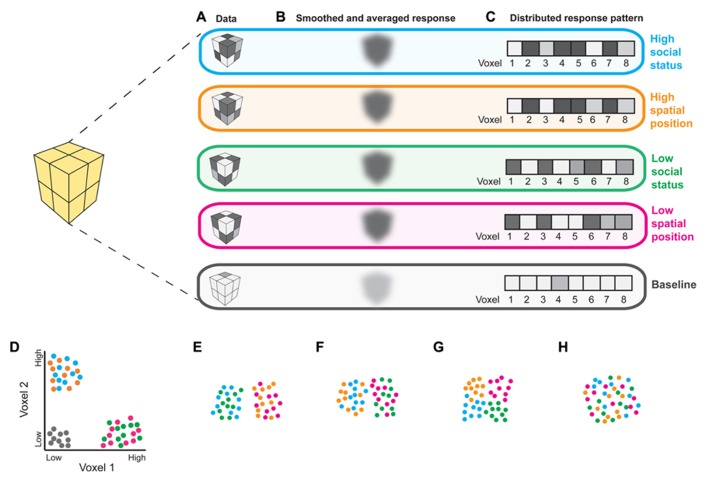
**Interpreting fMRI responses to social and spatial tasks.** **(A)** Hypothetical responses from an 8-voxel region of interest (ROI) to stimuli depicting: high social status (blue), high spatial position (orange), low social status (green), and low spatial position (pink), as well as baseline (gray; fixation cross). **(B)** Comparing the magnitude of locally smoothed and averaged responses could reveal that this ROI responds robustly to all 4 conditions relative to baseline, suggesting that it is involved in both social and spatial processing. **(C)** The same data can be studied as multivoxel patterns; responses from an ROI containing *n* voxels can be analyzed as *n*-dimensional vectors. Examining response patterns using MVPA can reveal more detailed information regarding the representational content of an ROI, as illustrated in **(D–H)**. **(D)** Responses from voxels 1 and 2 from the patterns depicted in **(C)** for 10 examples of each stimulus category; two-dimensional patterns are presented for clarity of visualization. Each dot represents a response to an example of each experimental condition. Experimental conditions are indicated by dot color. Machine learning algorithms can be used to determine which distinctions a region contains information about (Norman et al., 2006). Here, a linear classifier would accurately distinguish the 4 experimental conditions from baseline, as well as “high” social status and spatial position from “low” social status and spatial position, as would be expected from a brain region that represents social status analogously to spatial position. **(E–H)** Visualizations of possible representational similarity structures (Kriegeskorte et al., 2008) for responses that may not differ in average magnitude, as in **(B)**. Pairwise correlation distances between response patterns can be used to characterize pattern dissimilarity. Shorter distances between dots indicate greater pattern similarity; larger distances indicate greater pattern dissimilarity. Local response patterns within a region that is recruited for all 4 experimental conditions can contain information about domain (i.e., social vs. spatial) regardless of position (i.e., high vs. low spatial location or social status; **E**), position but not domain **(F)**, or about both domain and position **(G)**. Alternatively, such a region may not contain information useful in distinguishing either position or domain **(H)**. Thus, MVPA will be useful in testing whether overlapping fMRI activations for social and spatial tasks reflect shared or distinct processing mechanisms.

Further research is needed to characterize the relationship between the PPC’s involvement in social and spatial cognition. For instance, the TPJ is recruited both when subjects reason about others’false beliefs and positions in space (Abraham et al., 2008), suggesting that this region may perform similar computations on visuospatial and social contents. MVPA could be used to more directly test this possibility. Similarly, judgments about hierarchy and social distance recruit areas of the PPC involved in self-referential physical distance processing (Chiao et al., 2009; Yamakawa et al., 2009). Does this brain region represent “high” social status and “close” social distances analogously to how it represents “high” spatial location and “close” spatial distances? Again, characterizing the representational structure of the PPC with MVPA could elucidate this question (**Figure 1**).

Neuropsychological data also suggests a close relationship between representations of spatial and abstract information in the PPC. Patients with left hemineglect following right PPC damage often also neglect the “left” side of the mental number line (Zorzi et al., 2002), whereas PPC lesion patients without neglect show no numerical deficits (Vuilleumier et al., 2004). Remarkably, normal numerical processing is restored in neglect patients following interventions utilizing adaptation to leftward-shifting prism glasses that restore visual attention to the previously neglected side of space (Rossetti et al., 2004). Patients with hemispatial neglect exhibit analogous distortions of temporal processing, systematically overestimating temporal durations (Basso et al., 1996; Calabria et al., 2011). Spatiotemporal mappings appear to be supported by the PPC in healthy individuals, as they are diminished following transcranial magnetic stimulation to this region (Oliveri et al., 2009). Neuropsychological studies relating spatial and abstract cognition have focused primarily on non-social domains of abstract cognition (e.g., time, number) and space. However, Samson et al. (2004) reported impaired mentalizing in patients with focal lesions to the inferior PPC. To our knowledge, no studies have tested if PPC damage is associated with abnormal representations of one’s social network.

One limitation of neuroscientific evidence relating space and other domains of cognition is that data are available only from individuals in industrialized societies, and many of the corresponding behavioral phenomena are malleable to cultural learning (Dehaene et al., 1993). Although the tendency to map various domains of knowledge onto spatial representations appears to comprise a universal intuition (Dehaene et al., 2008; Parkinson et al., 2012), the nature of these mappings is often subject to cultural variation (Hung et al., 2008; Boroditsky and Gaby, 2010). Even two weeks of tool use engenders white and gray matter changes in the macaque PPC (Hubbard et al., 2005; Iriki, 2005). Lifelong immersion in cultures emphasizing metaphors and analogical reasoning no doubt impacts neural representations. Although the work summarized here is drawn from studies conducted in several countries, more cross-cultural work, especially that involving direct cross-cultural comparisons, is required to better understand how representational overlap between spatial and social cognition arises in the brain.

## COMPARING SPATIAL REPRESENTATIONS BETWEEN DOMAINS OF KNOWLEDGE

Importantly, although multiple domains of abstract cognition appear to co-opt mechanisms for spatial processing, different exaptations could have arisen separately, and may operate differently. There is a paucity of research investigating how different domains of knowledge that use space as a “reference domain” relate to one another. Different processes may have independently come to co-opt circuitry originally for spatial computations because such an arrangement was efficient and likely given pre-existing anatomical and functional constraints (Cantlon et al., 2009). Consistent with this suggestion, a recent study comparing spatial representations of number and pitch within individuals suggests that spatial representations are idiosyncratic to specific domains of knowledge (Beecham et al., 2009). Thus, although past work relating spatial cognition to non-social aspects of abstract cognition will be informative for future studies aimed at characterizing the relationship between spatial perception and social cognition, this will not be a trivial endeavor.

## CONCLUSION

Convergent evidence from behavior, neuropsychology, and neuroimaging suggest that humans use knowledge about space to scaffold mental representations of abstract information. Whereas most investigations have focused on non-social domains of abstract cognition, less work has explored the relationship between abstract aspects of social cognition (e.g., social distance evaluation, mentalizing) and spatial perception. Given the substantial progress that has stemmed from using this approach to characterize the mechanisms that support non-social domains of abstract cognition, we predict that relating abstract social cognition to spatial perception will be similarly fruitful. Further, given the centrality of sociality to human health and brain evolution (Dunbar, 1998), better understanding the mechanisms involved in social cognition is essential to understanding the human brain more generally.

## Conflict of Interest Statement

The authors declare that the research was conducted in the absence of any commercial or financial relationships that could be construed as a potential conflict of interest.
